# The impact of patient delirium in the intensive care unit: patterns of anxiety symptoms in family caregivers

**DOI:** 10.1186/s12913-021-07218-1

**Published:** 2021-11-05

**Authors:** Therese G. Poulin, Karla D. Krewulak, Brianna K. Rosgen, Henry T. Stelfox, Kirsten M. Fiest, Stephana J. Moss

**Affiliations:** 1 Department of Critical Care Medicine, Cumming School of Medicine, University of Calgary, Calgary, AB T2N 1N4 Canada; 2 Department of Critical Care Medicine, Cumming School of Medicine, University of Calgary, Calgary, AB T2N 1N4 Canada; 3 Departments of Community Health Sciences and Critical Care Medicine, Cumming School of Medicine, University of Calgary, Calgary, AB T2N 1N4 Canada; 4 Departments of Community Health Sciences and Critical Care Medicine, Cumming School of Medicine, University of Calgary, Calgary, AB T2N 1N4 Canada; 5 Departments of Critical Care Medicine, Community Health Sciences & Psychiatry, Cumming School of Medicine, University of Calgary, Calgary, AB T2N 1N4 Canada; 6 Departments of Community Health Sciences and Critical Care Medicine, Cumming School of Medicine, University of Calgary, Calgary, AB T2N 1N4 Canada

**Keywords:** Critical care, Delirium, Family, Anxiety, Intensive care unit, Engagement

## Abstract

**Background:**

The purpose of this study was to examine the association of patient delirium in the intensive care unit (ICU) with patterns of anxiety symptoms in family caregivers when delirium was determined by clinical assessment and family-administered delirium detection.

**Methods:**

In this cross-sectional study, consecutive adult patients anticipated to remain in the ICU for longer than 24 h were eligible for participation given at least one present family caregiver (e.g., spouse, friend) provided informed consent (to be enrolled as a dyad) and were eligible for delirium detection (i.e., Richmond Agitation-Sedation Scale score ≥ − 3). Generalized Anxiety Disorder-7 (GAD-7) was used to assess self-reported symptoms of anxiety. Clinical assessment (Confusion Assessment Method for ICU, CAM-ICU) and family-administered delirium detection (Sour Seven) were completed once daily for up to five days.

**Results:**

We included 147 family caregivers; the mean age was 54.3 years (standard deviation [SD] 14.3 years) and 74% (*n* = 129) were female. Fifty (34% [95% confidence interval [CI] 26.4–42.2]) caregivers experienced clinically significant symptoms of anxiety (median GAD-7 score 16.0 [interquartile range 6]). The most prevalent symptoms of anxiety were “Feeling nervous, anxious or on edge” (96.0% [95%CI 85.2–99.0]); “Not being able to stop or control worrying” (88.0% [95%CI 75.6–94.5]; “Worrying too much about different things” and “Feeling afraid as if something awful might happen” (84.0% [95%CI 71.0–91.8], for both). Family caregivers of critically ill adults with delirium were significantly more likely to report “Worrying too much about different things” more than half of the time (CAM-ICU, Odds Ratio [OR] 2.27 [95%CI 1.04–4.91]; Sour Seven, OR 2.28 [95%CI 1.00–5.23]).

**Conclusions:**

Family caregivers of critically ill adults with delirium frequently experience clinically significant anxiety and are significantly more likely to report frequently worrying too much about different things. Future work is needed to develop mental health interventions for the diversity of anxiety symptoms experienced by family members of critically ill patients.

**Trial registration:**

This study is registered on ClinicalTrials.gov (https://clinicaltrials.gov/ct2/show/NCT03379129).

**Supplementary Information:**

The online version contains supplementary material available at 10.1186/s12913-021-07218-1.

## Background

Critical illness is defined as an illness that is life-altering or life-threatening [1]. Critically ill patients are admitted to the intensive care unit (ICU) when they are fighting for their lives as these patients have complicated medical problems that require urgent treatment with life-sustaining technologies [2]. Critically ill patients in the ICU are among the sickest patients in in the healthcare system and caring for them is costly; ICU care accounts for 0.5–1% of the GDP [3].

Delirium occurs frequently among ICU patients [4], a serious and distressing neuropsychiatric syndrome with acute onset that fluctuates throughout the day [5]. Despite high prevalence of delirium in the ICU (estimates reported up to 80% in mechanically ventilated patients) [6, 7], delirium is often underdiagnosed and undertreated [8]. Screening vulnerable ICU patients for delirium is important for timely implementation of prevention and management measures [9].

Apart from the patient, family, or informal caregivers (i.e., relatives, friends) of critically ill patients are the only constant in the care journey. Family caregivers are essential members of the ICU team who often act as surrogate decision makers and important emotional supports during and after critical illness [10]. Family caregivers are not passive bystanders—they may recognize subtle changes in a patient first, provide a locus of familiarity for the patient, are important in improving processes of care associated with ICU transitions (to the hospital ward or to the community), and often act as advocates for the patient regarding treatment decisions [11].

Patients with delirium are often unable to communicate [12], which results in high levels of distress [13] and negative emotions in family caregivers of critical ill patients with delirium [14]. Highly distressed family caregivers may experience a breakdown in their relationship with the patient and may experience feelings of helplessness in relation to how to support their loved one [15]. A systematic review by Finucane and colleagues that consolidated experiences of family caregivers of terminally ill patients with delirium found that high levels of distress are experiences by caregivers of patients with delirium; reducing family caregiver distress and anxiety should be an important goal [16]. To inform future interventions the objective of this study was to examine the association of patient delirium in the ICU with patterns of anxiety symptoms in family caregivers when delirium was determined by clinical assessment and family-administered delirium detection [17, 18]. We hypothesized that family caregivers of critically ill patients with delirium would exhibit clinically significant patterns of anxiety symptoms [19–21].

## Methods

### Participants

**Fig. 1:**
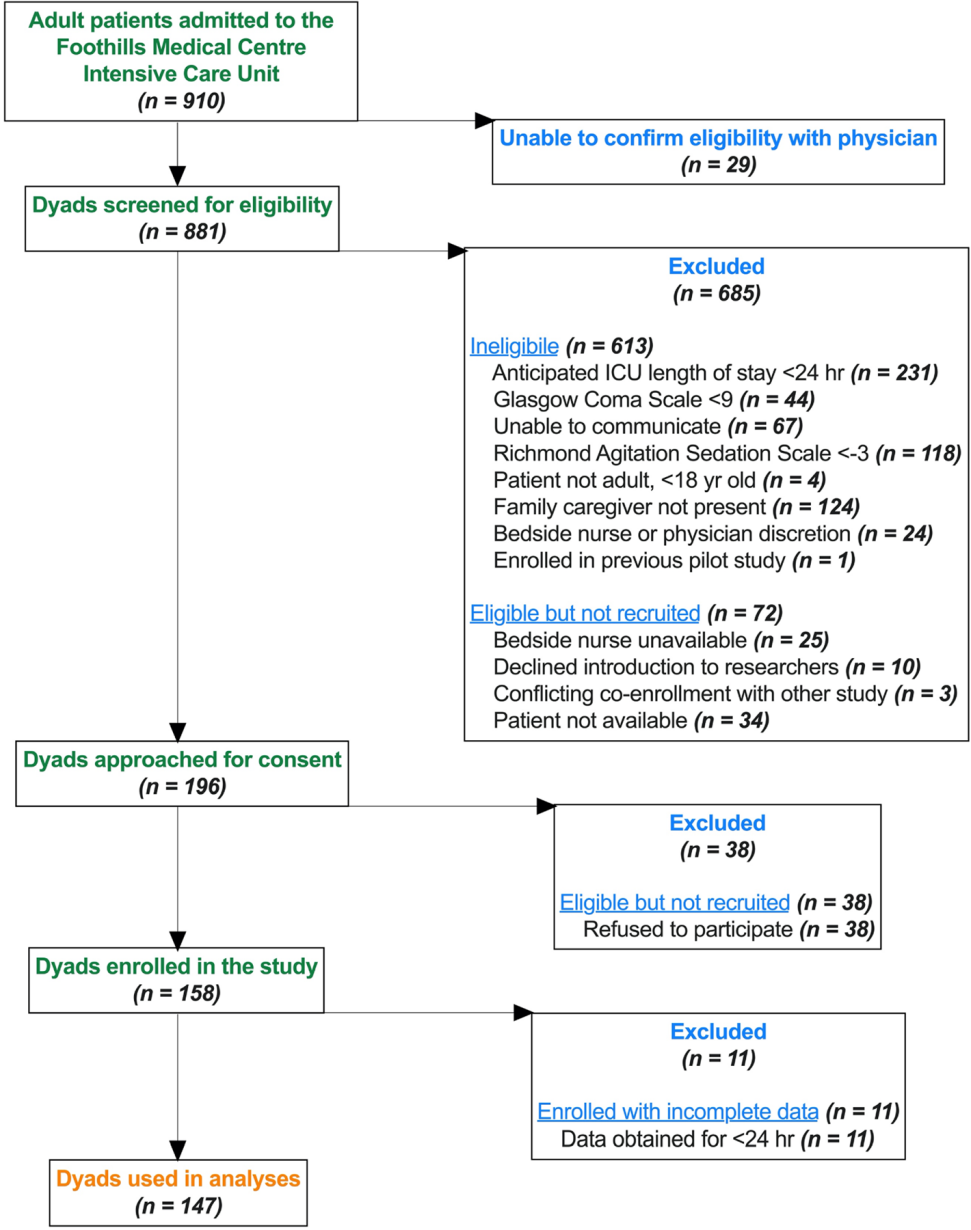
Study Flow Diagram

Between December 2017 and March 2019, 910 adult patients were admitted to the FMC ICU (Fig. 1). One hundred ninety-six dyads were approached for consent (out of 881 screened), of which 158 dyads were enrolled in the study; at least 24 h of data was obtained for 147/158 dyads (93%) [18] to achieve 95% sensitivity and 75% specificity, with a 10% margin of error and 80% power, estimating that 60% of critically ill patients would develop delirium [22]. Patient-family dyads were recruited from the Foothills Medical Centre (FMC, Calgary, Canada); a single-centre large academic hospital in a single-payer healthcare system (28 closed beds). Eligibility for participation (Table 1) was assessed daily by a trained research assistant granted approval from the bedside nurse. Consecutive eligible patients with at least one present family caregiver who provided informed consent were enrolled in the study as a patient-family dyad.

**Table 1:** Study Eligibility Criteria

Inclusion criteria	
Age 18 years or older Family member present Richmond Agitation-Sedation Scale score ≥ 3 (eligible for delirium detection)	
**Exclusion criteria**	
Patient or family did not provide informed consent Unable to community with research staff (e.g., hearing impairment, not fluent in English) Anticipated to have an ICU length of stay < 24 h New primary neurologic injury (e.g., severe traumatic brain injury) Glasgow Coma Scale score < 9	

### Procedure

Data were collected on eligible patients in the ICU up to a maximum of five days. Patient and family caregiver demographics were collected at first assessment. Patient clinical characteristics (e.g., Acute Physiology and Chronic Health Evaluation-II [APACHE-II]) were obtained from a beside clinical information database [eCritical] previously validated for research purposes [23]. Approval from the Conjoint Health Research Ethics Board at the University of Calgary was granted (REB 16–2060).

### Measures

#### Clinical delirium detection

Clinical assessment of patient delirium was conducted twice daily by a trained research assistant using the Confusion Assessment Method-ICU (CAM-ICU), a four-item dichotomous (i.e., delirium present, delirium absent) ICU delirium detection tool with published sensitivity (range: 69–82%) and specificity (range: 78–87%) in this sample [18].

#### Family-administered delirium detection

Family assessment of patient delirium (i.e., Sour Seven) was conducted once daily up to a maximum of five days. Family caregivers were blinded to results from clinical delirium assessments (and vice versa). In the present study, family caregivers assessed patient delirium using the Sour Seven, a family assessment of patient delirium symptoms related to altered awareness, disordered thinking, and reduced attention [24]. The Sour Seven was scored out of 18 with a cutpoint of ≥4 (i.e., probable delirium); in this cohort scores ≥4 have 73% sensitivity and 69% specificity [18]. This tool is administered easily without direct patient query [24].

#### Generalized anxiety disorder

Symptoms of generalized anxiety disorder among family caregivers were assessed with the Generalized Anxiety Disorder-7 (GAD-7) scale [25]. The GAD-7 is a self-report assessment of 7-items which measure GAD symptoms within the past two weeks using a four-point Likert-type scale (0=“Not at all” to 3=“Nearly every day” [range:0–21]). A cutpoint score of 10 (of 21) was used to indicate clinically significant GAD [26] with 89% sensitivity, 82% specificity [27]. In this study GAD symptom severity subgroups were scored as 0–5 = none; 6–19 = mild; 11–15 = moderate; and 16–21 = severe [25].

### Study design

This cross-sectional study is reported according to Strengthening the Reporting of Observational Studies in Epidemiology (STROBE) guidelines (Supplemental Table 1).

### Data analysis

Data are presented as numbers/percentage, mean or median, and compared statistically by *t*-tests or tests of proportions as appropriate. Prevalence estimates are reported with accompanying 95% confidence intervals (CIs). For the Sour Seven and GAD-7, the single most severe score or the first score (if all scores equally severe) were used in statistical analyses for each patient-family dyad [28]. No imputation techniques were used to handle missing data. Separate ordinal logistic regression analyses were computed for all seven items in the GAD-7 to determine the odds of scoring ≥2 (i.e., self-reported symptoms experienced more than half of the days) on each item based on patient delirium status regarding individual delirium detection assessment tools. Models were adjusted for family member age (dichotomized at 65 years [29, 30]), family member sex, family member education (high school or less, university/college), and patient APACHE-II score. Statistical analyses were conducted in STATA ICV.16 (StataCorp. College Station, TX: StataCorp LLC) and all *p*-values correspond to 2-tailed tests; *p* < 0.05 denotes significance.

## Results

Mean age of patients was 56.1 years (standard deviation [SD] 16.2) and 58 (39.5%) patients were female (Table 2). Nearly half of patients (*n* = 67, 45.6%) were admitted with a medical diagnosis and a median APACHE-II score of 20 (interquartile range [IQR], 12). As detected by the family-administered Sour Seven, 64.6% (95%CI 56.5–72.0) of critically ill patients experienced delirium at least once during their ICU stay, which was significantly higher compared to CAM-ICU clinical assessments (35.5, 95%CI 28.0–43.5; *p* < 0.001) (Supplemental Table 2).

**Table 2:** Demographic and Clinical Characteristics of Included Patients and Family Caregivers

Characteristic	Patient	Family Caregiver^**e,f**^				
		Clinically Significant Anxiety^**g**^	No Anxiety^**h**^	Mild Anxiety^**h**^	Moderate Anxiety^**h**^	Severe Anxiety^**h**^
*N (%)*	147 (100)	50 (34.0)	66 (44.9)	34 (23.1)	18 (12.2)	29 (19.7)
Age, yr, mean (SD)^a^	56.1 (16.2)	54.5 (13.3)	55.6 (14.0)	51.6 (14.7)	61.6 (11.0)	49.7 (12.5)
Sex, female, *n* (%)	58 (39.5)	45 (90.0)	45 (68.2)	21 (61.8)	14 (77.8)	28 (96.7)
Education, *n* (%)^b^						
High school or less	72 (49.7)	17 (34.0)	23 (34.9)	12 (35.3)	8 (44.4)	8 (27.6)
Some university/college or greater	73 (50.3)	33 (66.0)	43 (65.2)	22 (64.7)	10 (55.6)	21 (72.4)
Patient admitting diagnosis category, *n* (%)						
Medical	67 (45.6)	23 (46.0)	32 (48.5)	14 (41.2)	11 (61.1)	11 (37.9)
Neurologic	31 (21.1)	12 (24.0)	12 (18.2)	9 (26.5)	3 (16.7)	7 (24.1)
Trauma	27 (18.4)	9 (18.0)	11 (16.7)	6 (17.5)	3 (15.6)	6 (20.7)
Surgical	22 (18.4)	6 (12.0)	11 (16.7)	5 (14.7)	1 (5.6)	5 (17.2)
Acute Physiology and Chronic Health Evaluation II score, median (IQR)	20 (12)	18 (10)	21 (13)	19 (10)	21 (10)	17 (11)
Analgo-sedative use, *n* (%)	120 (81.6)	42 (84.0)	50 (75.8)	30 (88.2)	13 (72.2)	27 (93.1)
Patient Delirium, *n* (%)						
CAM-ICU^c^	52 (35.4)	21 (40.4)	19 (36.5)	14 (26.9)	5 (9.62)	14 (26.9)
Sour Seven^d^	95 (64.6)	34 (35.8)	36 (37.9)	28 (29.5)	7 (7.37)	24 (25.3)
Generalized Anxiety Disorder-7 score	–					
Median (IQR)	–	16 (6)	3 (3)	7 (2)	13 (3)	19 (3)

IQR = interquartile range

SD = standard deviation

GAD-7 = Generalized Anxiety Disorder-7

^a^One missing family member age

^b^Two missing patient education and one missing family education

^c^Scored as present/absent

^d^Sour Seven is scored out of 18; cutpoint of 4

^e^None missing family anxiety assessment

^f^Assessed by the Generalized Anxiety Disorder-7

^g^Scores 10 and above indicate clinically significant condition

^h^Scored as 0–5 = none; 6–19 = mild; 11–15 = moderate; 16–21 = severe

Dashes indicate no data to report for that group

### Prevalence of anxiety symptoms in family caregivers of critically ill patients

Among 34.0% (*n* = 50/147) of family caregivers with clinically significant anxiety symptoms [GAD-7 ≥ 10/21] (median GAD-7 score 16.0 [IQR 6]), mean age was 54.5 years (SD 13.3), of which 90% (*n* = 45) were female with at least some university or college education (*n* = 33, 66.0%).

When anxiety symptoms were stratified by level of severity, we found that most family caregivers frequently had none (*n* = 66, 44.9%) or mild (*n* = 34, 23.1%) symptoms of anxiety among which the majority were female (none, 45/66, 68.2%; mild, 21/34, 61.8%) and with higher education (none, 43/66, 65.2%; mild, 22/34, 64.7%). Many family caregivers self-reported severe (*n* = 29, 19.7%) symptoms of anxiety. Family caregivers with severe symptoms of anxiety were mostly female (96.7%) and younger (mean age 49.7 years [SD 12.5]) compared to family caregivers in other anxiety symptom severity subgroups. Overall, relatively fewer family caregivers (*n* = 18, 12.2%) reported moderate severity symptoms.

Most prevalent symptoms of anxiety experienced by family caregivers of critically ill patients more than half of the days (i.e., item score 2 or greater) were: Item 1, “Feeling nervous, anxious or on edge” and Item 2, “Not being able to stop or control worrying.” Item-level prevalence estimates for all included family caregivers are shown in Supplemental Table 3.

### Prevalence of anxiety symptoms in family caregivers of critically ill delirium patients

Estimated prevalence of self-reported clinically significant symptoms of anxiety among family caregivers of critically ill patients with delirium assessed by the CAM-ICU was 40.4% (95%CI 27.9–54.2) (Table 3). When patient delirium was detected by the family-administered Sour Seven compared to the clinical CAM-ICU tool, prevalence of clinically significant symptoms of anxiety among family caregivers was not significantly different (35.8, 95%CI 36.7–46.0; *p* = 0.58). Most prevalent symptoms of anxiety experienced by family caregivers of critically ill patients with delirium more than half of the days were: Item 1, “Feeling nervous, anxious or on edge” (Sour Seven, 44.2% [95%CI 34.5–54.4]); Item 2, “Not being able to stop or control worrying” (CAM-ICU, 44.2% [95%CI 31.4–57.9]; Sour Seven, 41.4% [95%CI 31.6–51.3]); Item 3, “Worrying too much about different things” (CAM-ICU, 44.2% [95%CI 31.4–57.9]); and Item 7, “Feeling afraid as if something awful might happen” (CAM-ICU, 46.2% [95%CI 33.1–59.8]; Sour Seven, 41.1% [95%CI 31.6–51.3]).

**Table 3:** Anxiety Prevalence for GAD-7 Items by Patient Delirium Detection

GAD-7 Items^**a**^	Clinical Assessment CAM-ICU^**c**^	Family-Administered Sour Seven^**d**^
Feeling nervous, anxious or on edge	42.3 (29.6–56.1)	**44.2 (34.5–54.4)**
Not being able to stop or control worrying	**44.2 (31.4–57.9)**	**41.4 (31.6–51.3)**
Worrying too much about different things	**44.2 (31.4–57.9)**	36.8 (27.7–47.0)
Trouble relaxing	38.5 (26.2–52.3)	36.8 (27.7–47.0)
Being so restless that it is hard to sit still	28.8 (18.1–42.6)	24.2 (16.6–33.9)
Becoming easily annoyed or irritable	30.8 (19.7–44.6)	26.3 (18.4–36.1)
Feeling afraid as if something awful might happen	**46.2 (33.1–59.8)**	**41.1 (31.6–51.3)**
Total Score^b^	40.4 (27.9–54.2)	35.8 (26.7–46.0)

CAM-ICU = Confusion Assessment Method for ICU

GAD-7 = Generalized Anxiety Disorder-7

^a^Each item scored as 0, not at all; 1, several days; 2, more than half the days; 3, nearly every day

^b^Total score 10 and above indicates clinically significant condition

^c^Scored as present/absent

^d^Sour Seven is scored out of 18; cutpoint of 4

The prevalence of family caregivers for critically ill patients with delirium to indicate an item score 2 or greater or to report a total score that indicates clinically significant anxiety

All values represent % with 95% CIs

**Bold text** indicates most prevalent symptoms of anxiety

### Associations of family caregiver anxiety symptoms

**Fig. 2:**
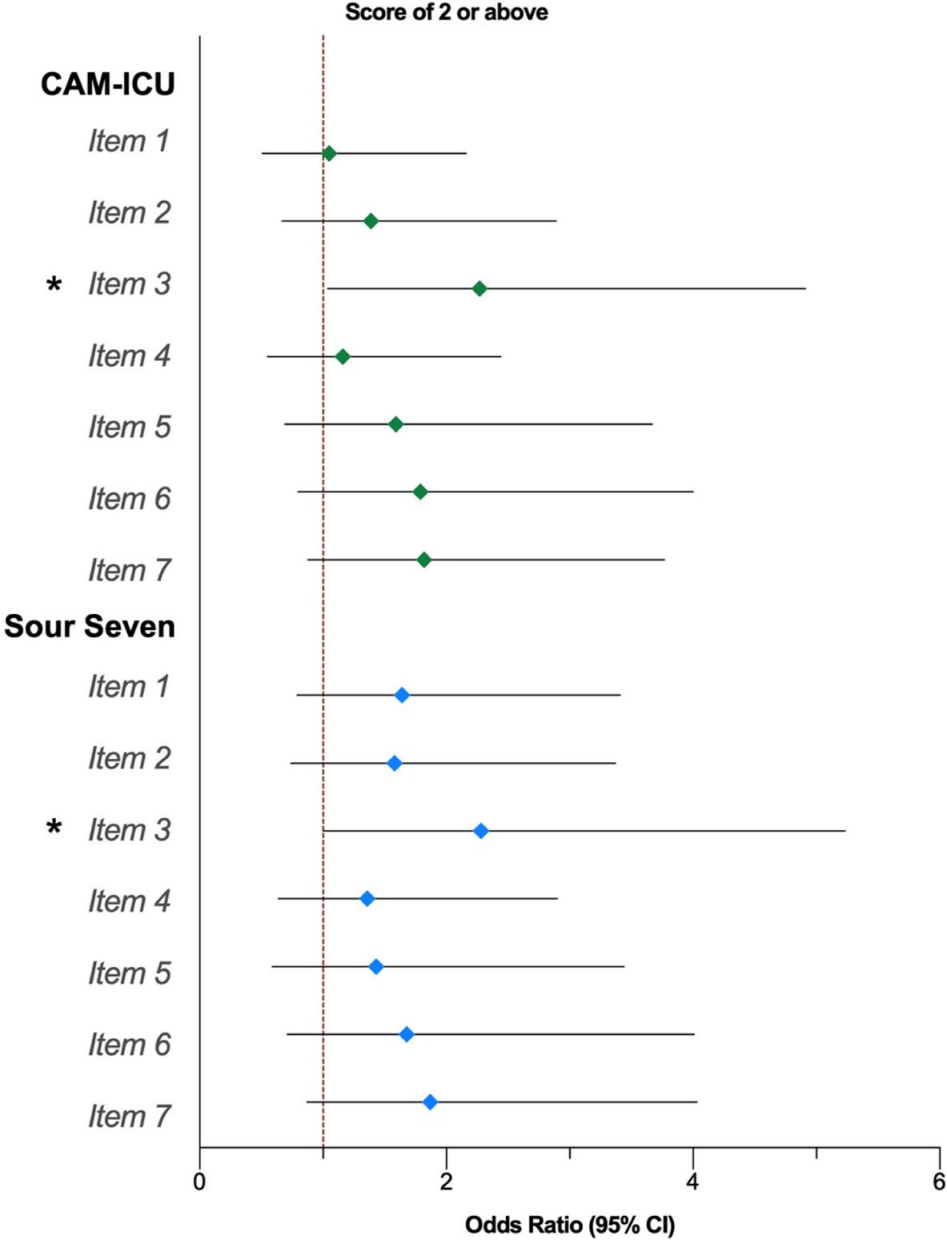
Ordinal Logistic Regression Analyses for GAD-7 Items by Patient Delirium Detection Tool. GAD-7 = Generalized Anxiety Disorder-7. Each item scored as 0, not at all; 1, several days; 2, more than half the days; 3, nearly every day. Sour Seven is scored out of 18; cutpoint of 4. Ordinal logistic regression (cumulative logit) of ordered GAD-7 items; Odds ratio above 1 indicate trend towards item score 2 or greater among family caregivers of patients with delirium. Models adjusted for family age, family sex, family education, and patient Acute Physiology and Chronic Health Evaluation II score (dichotomized at median value). All values represent odds ratio with 95% CI; ^*^ = *p* < 0.05

Figure 2 illustrates self-reported anxiety symptoms in family members of patients with and without delirium by patient delirium detection tool; results are presented in Table 4, with values adjusted for family age, family sex, family education and dichotomized (at median) patient APACHE-II score. Family caregivers of critically ill patients with delirium scored significantly higher than family caregivers of critically ill patients without delirium on item 3 regarding “Worrying too much about different things” (CAM-ICU, OR 2.27 [95%CI 1.04–4.91]; Sour Seven, OR 2.28 [95%CI 1.00–5.23]).

**Table 4:** Ordinal Logistic Regression Analyses for GAD-7 Items by Patient Delirium Detection

GAD-7 Items^**a**^	Clinical Assessment CAM-ICU^**b**^	***p***-value	Family-Administered Sour Seven^**c**^	***p***-value
Feeling nervous, anxious or on edge	1.05 (0.51–2.16)	0.89	1.64 (0.79–3.41)	0.19
Not being able to stop or control worrying	1.39 (0.67–2.89)	0.38	1.58 (0.74–3.37)	0.24
Worrying too much about different things	2.27 (1.04–4.91)	0.04	2.28 (1.00–5.23)	0.05
Trouble relaxing	1.16 (0.55–2.44)	0.69	1.36 (0.64–2.90)	0.43
Being so restless that it is hard to sit still	1.59 (0.69–3.67)	0.28	1.43 (0.59–3.44)	0.42
Becoming easily annoyed or irritable	1.79 (0.80–4.00)	0.15	1.68 (0.71–4.01)	0.24
Feeling afraid as if something awful might happen	1.82 (0.88–3.77)	0.11	1.87 (0.87–4.03)	0.11

CAM-ICU = Confusion Assessment Method for ICU

GAD-7 = Generalized Anxiety Disorder-7

^a^Each item scored as 0, not at all; 1, several days; 2, more than half the days; 3, nearly every day

^b^Scored as present/absent

^c^Sour Seven is scored out of 18; cutpoint of 4

Ordinal logistic regression (cumulative logit) of ordered GAD-7 items; Odds ratio above 1 indicate trend towards item score 2 or greater among family caregivers of patients with delirium

Models adjusted for family age, family sex, family education, and patient Acute Physiology and Chronic Health Evaluation II score (dichotomized at median value)

All values represent odds ratio with 95% CIs

## Discussion

In this observational study we sought to examine the association of patient delirium in the ICU with patterns of anxiety symptoms in family caregivers when delirium was determined by clinical assessment and family-administered delirium detection. We hypothesized that family caregivers of critically ill patients with delirium would exhibit clinically significant patterns of anxiety symptoms [19–21]. Our study has two main findings. First, family caregivers of critically ill patients with delirium (compared to those without delirium) were more likely to report “Worrying too much about different things” more than half of the time. This may imply that delirium in critically ill patients negatively affects family caregivers and may increase the likelihood of experiencing anxiety symptoms associated with GAD, particularly worrying about too many things. Second, we found no significant difference between clinically significant symptoms of anxiety in family caregivers of critically ill patients with delirium when delirium was assessed using a clinical or the family-administered tool. This may suggest that delirium assessed by a family caregiver had no effect on caregiver anxiety.

Consistent with previous studies [31–35], anxiety symptoms were highly prevalent in family caregivers of critically ill adult patients. We found overall mean scores for anxiety symptom severity subgroups were higher than those reported from primary caregivers of hospital-based individuals with mental illness [36], comparable to community-dwelling spouse or child caregivers for individuals with Alzheimer’s [37], which a group identified at particularly high risk for poor psychological and mental health outcomes [38, 39]. Notably, 34% of family caregivers reported mean GAD-7 scores above cutpoint for clinical significance and of these caregivers, 58% reported severe symptoms of anxiety.

Distinct characteristics of family caregivers who reported worse patterns of anxiety symptoms included being female and younger in age. Females are more likely to visit the ICU, be family caregivers, and spend more hours providing care [40–42]. Female caregivers are also more likely to not get enough sleep or regular physical activity [43], which indicates that females may be at increased risk for the harmful health effects of caregiver stress. Further, younger family caregivers compared to older family caregivers may face additional stressors such as financial burden and lifestyle interferences [44]. Younger caregivers may feel a disproportionate amount of emotional and physical strain that may exacerbate severity of anxiety symptoms. We also found that patient admitting diagnoses were varied and majority of patients were administered an analgesic together with a sedative. These results agree with what is reported in the literature; it is suggested the protective effects of caregiver male gender, increased caregiver age and less severe patient illness affects psychological functioning and coping with stress among family caregivers of the critically ill [45, 46]. As we asked a single global demographic question on age and gender and did not query family caregiver coping capabilities, our data do not permit us to address age and gender differences related to psychological coping—a complex multidimensional construct [47]—in explaining patterns of family caregiver anxiety symptoms. However, our findings are consistent with those reported by Bolosi and colleagues [48] and are in line with the notion generally [49] that younger, female family caregivers of more severely critically ill patients are at increased risk of developing more clinically pronounced symptoms of anxiety during a patient’s ICU stay.

Our results indicate that family caregivers of critically ill adults experience common symptoms of anxiety and that caregiving for a critically ill adult patient with delirium increases the likelihood of experiencing symptoms anxiety related to GAD. In the ICU, family caregivers are often present at bedside [50] and, playing a key role in their patients’ overall care, are commonly engaged to support the patient through non-pharmacological delirium management interventions [51]. Family caregivers of critically ill patients with delirium frequently report the experience of seeing their loved one suffering from delirium as distressing [15]. The negative psychological sequalae experienced by family caregivers unique to their loved one’s narrative of critical illness likely contributes to the pattern and severity of anxiety symptoms that they confront [52].

Several studies in various practice settings indicate that patient delirium is distressing to family caregivers [53–55]. Cross-sectional studies have reported that family caregivers of cancer patients with caregiver-detected delirium were significantly more likely to meet criteria for GAD [56]. Our previous work in this cohort found that caregiver-detected delirium score was associated with severity of family caregiver anxiety symptoms (coefficient 0.2, 95%CI 0.1–0.4) [57]. The present findings narrow down the association of family caregiver anxiety and ICU patient delirium to a single item on the GAD-7. It is possible that the relatively small sample size resulted in insufficient power to detect significant associations with other items on the GAD-7. Further study using designs that account for temporality (e.g., cohort study) on the relationship between caregiver anxiety and patient delirium to distinguish patterns of anxiety symptoms in explaining mechanisms related to anxiety and delirium (i.e., psychopathology) including moderators (e.g., psychopathology, depression) is warranted. This can help develop effective interventions targeted to specific patterns of anxiety to improve anxiety symptoms among family caregivers of the critically ill.

Engaging and empowering family caregivers in patient care may yield potential benefits for caregiver anxiety symptoms when risk factors for anxiety are incorporated in designing mental health strategies for improved psychological outcomes [58]. Highly resilient individuals are known to proactively cultivate positive adaptations [59]. Incorporating positive mental health strategies that promote for examples humanity (love, kindness) or transcendence (hope, spirituality) might allow family caregivers to adapt to the evolving demands and stress of the critical illness experience [60, 61]. These strategies could also improve positive psychological coping behaviors [62, 63] and the symptoms of anxiety, especially in female family caregivers. We recommend future mental health interventions targeted to family caregivers’ anxiety in the ICU consider both negative (e.g., anxiety) and positive (e.g., humanity, transcendence) psychological outcomes.

Our results require cautious interpretation. The cross-sectional nature of data acquisition meant we were unable to detect incident family caregiver anxiety or patient delirium. Enrollment and initial assessments of patient-family dyads occurred after ICU admission, thus no baseline data for anxiety symptoms or patient cognitive functioning were available. These results may not be generalizable to other populations given our study was conducted at a single-centre in a single-payer healthcare system; however, this tertiary care medical centre serves a catchment area of 1.8 million people. Family caregivers were not always present at bedside, making consecutive delirium and anxiety assessments over a standardized timeframe challenging. Considering family caregivers with less severe symptoms are more likely to engage in research [64], our study may have a potential selection bias for family caregivers with less severe symptoms of anxiety; therefore, the burden of anxiety might be greater than estimated. As well, family-administered delirium detection tools have lower diagnostic accuracy compared to clinical assessments of patient delirium, though their operating characteristics are fair [18] and pose a viable option when clinical assessments are not feasible [65].

## Conclusions

Family caregivers of critically ill adult patients often experience clinically significant symptoms of anxiety and are significantly more likely to report frequently worrying too much about different things when their loved one develops delirium. Our findings suggest delirium in critically ill patients negatively affects family caregivers to increase the likelihood of experiencing anxiety symptoms associated with GAD. Longitudinal studies of the patterns of anxiety in caregivers of critically ill patients with delirium is necessary to develop targeted treatments and interventions to improve anxiety symptoms.

## Supplementary Information

**Additional file 1.**
